# Macrodystrophia lipomatosa: Clinical and radiological insights into localized gigantism

**DOI:** 10.1016/j.radcr.2024.11.029

**Published:** 2024-12-07

**Authors:** Nishtha Manuja, Sandip Mohale, Shreya Khandelwal, Anshul Sood

**Affiliations:** a Department of General Medicine, Jawaharlal Nehru Medical College, Datta Meghe Institute of Higher Education and Research, Sawangi (Meghe), Wardha, Maharashtra, India, 442001; b Department of Radiodiagnosis, Jawaharlal Nehru Medical College, Datta Meghe Institute of Higher Education and Research, Sawangi (Meghe), Wardha, Maharashtra, India, 442001

**Keywords:** Macrodystrophia lipomatosa (MDL), Mesenchymal, Macrodactyly, Radiology

## Abstract

A rare type of localized gigantism known as macrodystrophia lipomatosa is characterized by a disproportionate increase in fibroadipose tissues and a gradual overgrowth of all mesenchymal elements. The distribution in the lower extremities’ plantar nerves and the upper extremity's median nerve is most commonly observed. This abnormality is congenital and typically manifests at birth or during the neonatal stage. This deformity begins to mechanically impair joint function, blood supply, and innervation as age advances. The findings from radiography include lucencies in the soft tissues and expansion of the digit's phalanges and soft tissue components, with predominantly distal component involvement. Herein, we present a case of a 20-year-old male from rural India who came to us with the complaint of abnormal asymmetrical swelling of bilateral hand fingers, which has been progressing since birth. Physical examination revealed a soft, non-fluctuant, non-pulsatile swelling with no associated trauma or injury. The clinical picture revealed disproportionate enlargement of phalanges in both hands.

## Introduction

Macrodystrophia lipomatosa is a rare congenital, nonhereditary condition characterized by localized gigantism involving any part of the body. In most cases, it affects digits and extremities with fibrolipomatous hamartoma (FLH) of the nerves [1,2]. The term macrodystrophia lipomatosa was first coined by Feriz in 1925 [3]. It is commonly described as lipofibromatous hamartoma (LFH) in association with macrodactyly, along with the involvement of all mesenchymal elements, it is often referred to as MDL [4]. It can be associated with syndactyly, polydactyly, or clinodactyly. The condition presents as painless enlargement of the second or third digit of the hand or foot. It usually affects digits in the distribution of the median nerve in the upper extremity. At times it can also involve an entire limb [5]. The clinical picture presents marked hypertrophy of the phalanges and the soft tissue shows hypertrophied adipose tissue of the involved digits. We still are not clear with the incidence [6]. The afflicted people typically experience a crippling functional and psychological impact on them and their families. The illness frequently results in mechanical problems, and cosmetic deformity is the primary cause of care.

This condition is treated with surgical intervention to enhance the cosmetic appearance while conserving neurologic function. In our case, bilateral involvement of the upper extremity with the involvement of multiple digits is seen.

## Case presentation

A 20-year-old male patient presented to the outpatient department (OPD) of Medicine complaining of unusual asymmetrical swelling in his right hand's little finger, ring finger and middle finger and left hand's little finger and ring finger. The swelling had been increasing since birth causing him discomfort and hampering his daily routine activities. When he initially noticed this swelling as a child, he didn't seek treatment because it didn't significantly interfere with his ability to perform his everyday tasks. Since childhood, the swelling was progressively increasing. There was no prior history of trauma or injury. There was no history of any comorbidities. There was no significant family history.

**Fig. 1:**
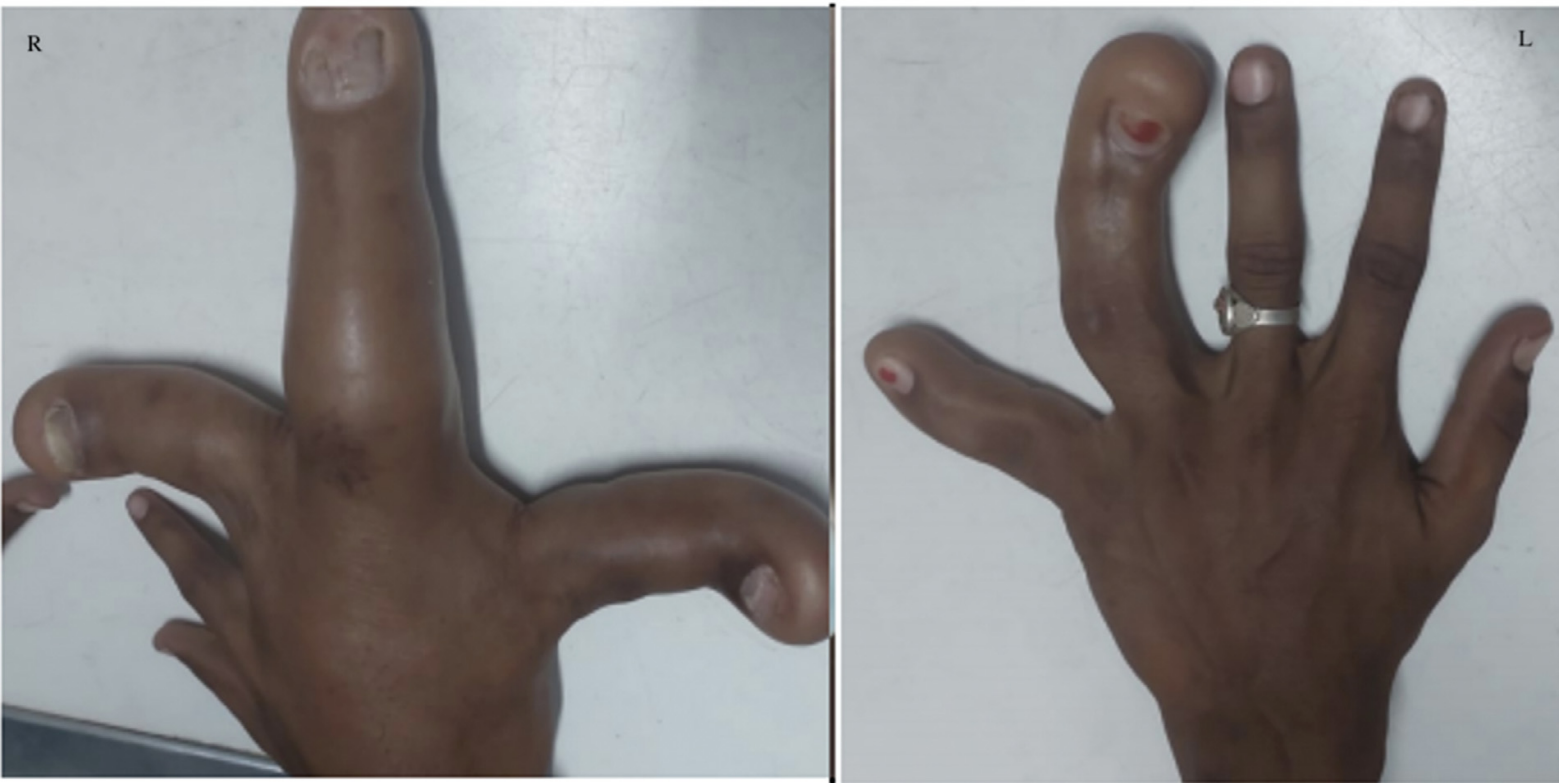
A clicked picture of the right and left hand showing asymmetrically enlarged multiple digits of the bilateral hand.

On physical examination, the swelling was localized to the third, fourth and fifth digits of the right hand and the fourth and fifth digits of the left hand. It was nontender, soft in consistency, non-fluctuant and non-pulsatile. There was no local erythema or any scar mark, as shown in Fig. 1. The affected digit's range of motion was restricted. No concomitant sensory loss was seen. The blood investigations were within normal limits.

**Fig. 2:**
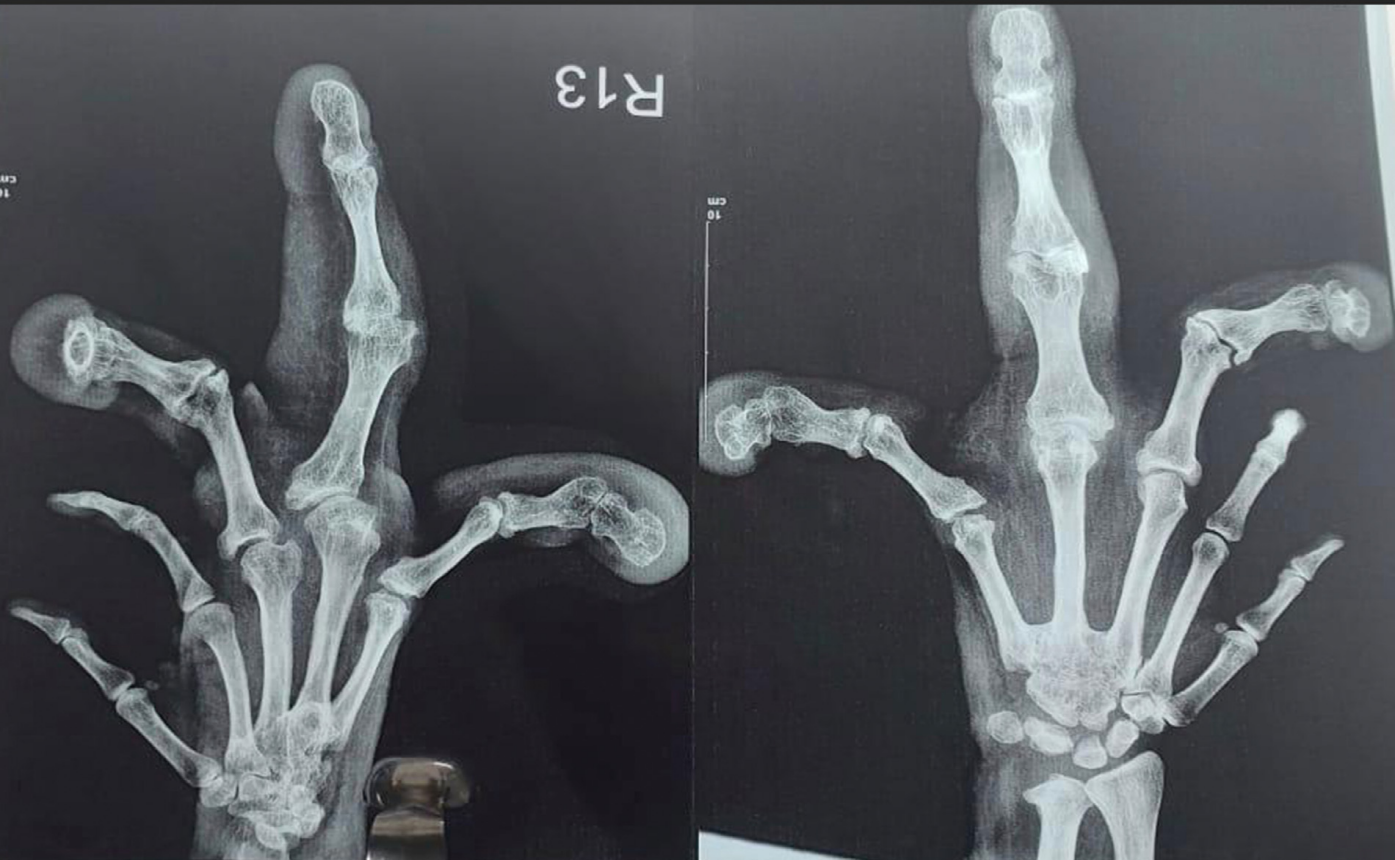
X-ray AP Oblique and PA view of Right hand showing abnormally enlarged third, fourth and fifth phalanges and metacarpals with hypertrophy of the soft tissues surrounding it.

Xray of the right hand revealed an enlarged third, fourth and fifth phalanx and metacarpals with a fusion of the base of third, fourth and fifth metacarpals along with a fusion of carpals- capitate and hamate and hypertrophy of the overlying soft tissue as shown in Fig. 2.

## Discussion

Macrodystrophia lipomatosa, a localized gigantism, is a non-hereditary congenital disorder affecting a small part of the limb/finger or the entire limb. It is characterized by enlargement of the fingers, due to an abnormal proliferation of fibroadipose tissue, fatty infiltration and hypertrophy of all components of the finger, including skin, bone, and nerves [7]. The etiology of macrodystrophia lipomatosa is still not clear, and recent studies have shown an association with PIK3CA gene mutation [8]. In our case, the patient had a fusion of the base of metacarpals along with two carpals, capitate and hamate, which has not been reported previously as per the published literature. However, the genetic testing was not done due to financial constraints.

This lesion is clinically present from birth and frequently detected throughout the first three years of life. There are two identified types: the static type, which causes continuous yet slow enlargement leading to cosmetic problems, and the progressive form, which tends to increase the fingers or extremities rapidly. Although it usually affects one finger, this condition can affect multiple fingers. The thumb involvement is characterized by abduction and extension and typically exhibits a radial curvature deformity. The involvement of two or more fingers usually leads to divergent deviation. Bone expansion occurs, and the deviation typically lasts until the closure of the epiphyseal plate. The soft tissue will continue getting bigger with age [9]. Some cases even present with the presence of syndactyly, clinodactyly, brachydactyly and polydactyly [5].

Pathologically, macrodystrophia lipomatosa shows an increase in all mesenchymal elements with excess adipose tissue together with other tissues such as periosteum, bone marrow, nerve sheath, muscle, and subcutaneous tissue [10].

Microscopic examination shows an increase in the quantity of subcutaneous fat, enlargement and excessive growth of nerve cells [11].

A radiological examination is essential for making a diagnosis of the illness. A plain radiograph displays increased adipose tissue-related soft tissue and bone hypertrophy. In addition to endosteal and periosteal bone deposition, the distal phalanges are lengthened and expanded into a trapezoid like structure. The volar and distal features are often affected by overgrown soft tissues.

The narrowing of the joint space, subchondral cysts and osteophytes are seen in adolescent or young adult patients which suggests forms of secondary osteoarthritis [12].

Ultrasound examination reveals tissue hypertrophy and nerve involvement, hypertrophy distribution of adipose tissue (subcutaneous, intermuscular or intramuscular), and calcification, abnormal blood flow due to vascular malformations which cannot be detected by plain X-ray [7].

A CT scan shows fat proliferation along the course of the peripheral nerves. A CT scan will more clearly reveal excessive bone growth in the area supplied by the peripheral nerves [13].

An MRI examination reveals fibrous tissue, an excess of adipose tissue, bone hypertrophy, and cortical thickness in the affected area of the body, which results in the affected bone exostosis.

Surgery is the most definitive management. Non-surgical management with observation and follow-up can be done if the enlargement is not too debilitating and there are no symptoms of peripheral nerve entrapment [14].

Indications for surgical management include compression of the nerves, functional impairment due to swelling of fingers or extremities, and cosmesis problems. Based on the patient's symptoms, age, and severity, the priority of surgery varies [15].

Surgical management involves debulking of soft tissue, primarily adipose tissue, which is followed by progressive repair, reduction osteotomy, carpal tunnel compression to release peripheral nerves, epiphysiodesis, or ray amputation. A large debulking procedure (30 to 50%) may result in complications, including neurological damage with a recurrence probability of 33% to 60%. The goal is to stop abnormal growth of limbs and correct deformities due to disproportionate growth by epiphysiodesis and osteotomy to achieve good results. Amputation is done when the affected limb or the fingers cannot function properly after therapy, and to prevent further complications such as nerve entrapment in the future [7].

## Conclusion

Surgical management in macrodactyly due to macrodystrophia lipomatosa of the finger gives a positive result. Surgical management results show a decrease in mass, lesser compression of the nerves, and decreased functional impairment due to enlarged fingers.

## Patient consent

An informed verbal and written consent was obtained from the patient.
